# Internet Gaming Disorder in Children and Adolescents with Autism Spectrum Disorder and Attention Deficit Hyperactivity Disorder

**DOI:** 10.3390/brainsci14020154

**Published:** 2024-02-02

**Authors:** Valerio Simonelli, Antonio Narzisi, Gianluca Sesso, Andrea Salvati, Annarita Milone, Valentina Viglione, Greta Tolomei, Gabriele Masi, Stefano Berloffa

**Affiliations:** 1 Department of Clinical and Experimental Medicine, University of Pisa, 56128 Pisa, Italy; valerio.simonelli@fsm.unipi.it; 2 IRCCS Stella Maris, Scientific Institute of Child Neurology and Psychiatry, Viale del Tirreno, 331A, Calambrone, 56025 Pisa, Italy; antonio.narzisi@fsm.unipi.it (A.N.); gianluca.sesso@fsm.unipi.it (G.S.); annarita.milone@fsm.unipi.it (A.M.); valentina.viglione@fsm.unipi.it (V.V.); greta.tolomei@fsm.unipi.it (G.T.); stefano.berloffa@fsm.unipi.it (S.B.); 3 Social and Affective Neuroscience Group, Molecular Mind Lab, IMT School for Advanced Studies Lucca, 55100 Lucca, Italy; 4 Child and Adolescent Psychiatry Unit, A. Meyer Children’s Hospital, Viale Pieraccini 24, 50139 Florence, Italy; andreasalv91@gmail.com

**Keywords:** internet gaming disorder, autism spectrum disorder, ADHD, behavioral addiction

## Abstract

Attention deficit hyperactivity disorder (ADHD) and autism spectrum disorder (ASD) have been related to an increased risk for behavioral addictions including online gaming. However, the relationship between these two conditions and Internet gaming disorder (IGD) is still debated. The aim of this study is to address this topic by exploring the prevalence of IGD in a consecutive sample of ASD youth and ADHD youth, compared with a normal control group, and by assessing selected psychopathological and neuropsychological features in ASD and ADHD patients with and without IGD. This study included 77 ASD patients (67 males, mean age 13.58 ± 2.75 years), 94 ADHD patients (79 males, mean age 11.46 ± 2.47 years), and 147 normal controls (NC) (mean age 13.9 ± 3.0 years, 114 males) that received structured measures for IGD (IAT, IGDS9-SF, and UADI). In the ADHD group, 72.34% of the sample were above the IGD cut-off, compared with 45.45% in the ASD group and 9.5% in the NC group. ASD patients with IGD presented with greater severity and more severe attention problems, with no difference in the ASD core symptoms between patients with and without IGD. In the comparison between the ASD and ADHD groups according to the presence of IGD, ASD patients with IGD were the most severe group according to the CGI (Clinical Global Impression) scale. The follow-up, conducted on 45 patients affected by ASD, showed an improvement in CGI and CGAS (Children’s Global Assessment Scale) scores, but not in the IGD symptoms. These findings could place the diagnosis of ASD as a negative prognostic factor in the follow-up of aspects of video game addiction compared with ADHD.

## 1. Introduction

Internet gaming disorder (IGD) has been included by the American Psychiatric Association (APA) in the fifth edition of the Diagnostic and Statistical Manual of Mental Disorders (DSM-5) [1] as a new condition that requires further research evidence. IGD has been defined as featured by a “persistent and recurrent” use of the Internet for gaming that leads to significant impairment and distress in several areas of functioning [1]. IGD currently refers to the specific dimension of pathological online gaming, although numerous other terms have been suggested in the literature to describe the pathological use of technological devices such as Internet addiction [2], problematic Internet use [3], Internet use disorder [4], smartphone addiction [5], etc. IGD is also under debate among clinicians and researchers since, even though the DSM-5 defined it as a separate diagnostic category, the evidence from the literature demonstrates its comorbid presence within several different psychiatric disorders. Therefore, this would lead to considering it as an epiphenomenon of other clinical conditions with which it is associated [4], making it difficult to find it as a single disorder in patients. Specifically, IGD appears to be often associated with conditions featured by social withdrawal [6], such as anxiety and depression [7], and it is plausible to hypothesize that Internet gaming represents a way of keeping social relationships that would be otherwise precluded and limited in real life [6], which also accounts for the frequent comorbidity with autism spectrum disorder (ASD) [8]. Furthermore, several studies in the literature have found associations between attention deficit and hyperactivity disorder (ADHD) and Internet gaming disorder [9,10], as well as for behavioral disorders closely connected to ADHD such as oppositional defiant disorder [11].

By definition, ASD is characterized by persistent deficits in social communication and social interaction in multiple contexts, which manifest themselves specifically in deficits in socio-emotional reciprocity. These are associated with restricted and repetitive patterns of behavior, interests, or activities [1]. A recent review [12] found a significant association between these two conditions. The main hypotheses regarding the association between IGD and ASD evaluated the use of video games as a tool for “facilitating” relationships with peers [13], as well as the possibility that the better visuospatial abilities typically found in autistic patients make them skilled in gaming, which requires identifying numerous stimuli in complex virtual environments [14]. However, many of the reported studies are based on adult patients and differences can be found in the use of standardized tests to investigate IGD [12].

ADHD instead is a condition characterized by a persistent pattern of inattention and/or hyperactivity–impulsivity that interferes with functioning or development [1]. It has been widely highlighted in the literature that ADHD can be a significant predictor of both substance use disorder [15] and behavioral addictions [16], as these disorders share neuropsychological characteristics with ADHD [17,18]. A recent systematic review summarized 29 studies evaluating the association between ADHD and gaming disorder, of which only 11 were based on clinical samples [19]. The review found a consistent positive association between ADHD and IGD, either in clinical-based or community-based samples, particularly for the inattention subscale. In contrast, hyperactivity was less commonly associated with IGD.

Although the literature has consistently found associations between IGD and both clinical ASD and ADHD, there are only a few studies on such associations in the pediatric population [12,19]. Furthermore, there is only one study that evaluated the IGD symptoms in both ASD and ADHD in a pediatric population [20]. According to this, the present study has the following objectives: (1) investigating the prevalence of IGD in a population of pediatric patients diagnosed with ASD and comparing it with that of a population of pediatric patients with ADHD and to a control sample; and (2) describing the psychopathological and neuropsychological profile of patients with ASD and IGD compared with ASD patients without IGD and comparing it with that of ADHD patients. Moreover, we aimed to longitudinally assess the effectiveness of clinical monitoring and pharmacological and/or psychotherapeutic treatment on the reduction in IGD symptoms in a population of patients suffering from ASD.

## 2. Materials and Methods

### 2.1. Sample and Recruitment

The clinical sample, composed of patients diagnosed with ASD and ADHD, was recruited between December 2020 and July 2023 at the IRCCS Stella Maris Foundation hospital. Overall, 171 subjects were recruited (age range 8–17 years), who were divided into two clinical groups: patients affected by ASD (77 patients, of which 67 were males, mean age 13.58 ± 2.75 years) and patients suffering from ADHD (94 patients, of which 79 were males, mean age 11.46 ± 2.47 years). The diagnosis was made according to the DSM-5 criteria, through clinical history and psychiatric assessment using the semi-structured clinical interview K-SADS-PL [21]. History, assessment, and clinical interviews were carried out by trainee residents under the supervision of a senior psychiatrist. Exclusion criteria were comorbidity with intellectual disability or psychotic disorder (due to inability to complete the questionnaires). The control group was recruited between December 2020 and May 2021 in the regions of Tuscany and Campania. The sample included 147 subjects, 114 males (78%), aged between 8 and 17 years (mean age 13.9 ± 3.0 years). A follow-up evaluation was carried out for 45 subjects affected by ASD after 4–6 months from the initial assessment (Supplementary Figure S1). Within the clinical group, ASD (Table 1) and ADHD patients (Table 2) exhibited the following comorbidities.

### 2.2. Procedures

All study participants, including those in the two clinical groups and those in the control group, completed standardized tests to assess Internet use and Internet gaming disorder, specifically using the Internet Addiction Test (IAT) [22] and the Internet Gaming Disorder Scale—Short Form (IGDS9-SF) questionnaires, respectively [23]. All study participants received a detailed explanation about the content of the questionnaires (orally for younger children and through written text for older children and adolescents). Children aged between 11 and 17 also completed the questionnaire on Internet use, abuse, and addiction (UADI) [24], aimed at qualitatively evaluating video addiction. The clinical sample was also administered a clinical severity rating scale, the Clinical Global Impression—Severity Scale (CGI-S) [25], and a global adaptive functioning scale, the Children’s Global Assessment Scale (C-GAS) [26]. Parents of patients with ASD completed the Social Responsiveness Scale-2 (SRS-2) [27], the Autism Quotient version for adolescents [28] and children [29] depending on the age range, the Social Communication Questionnaire (SCQ) [30], and the Systematizing Quotient (SQ) [31] for the evaluation of aspects of social responsiveness and the CBCL for a dimensional evaluation of the clinical features. Finally, the cognitive profile of the clinical group was assessed using the Wechsler Intelligence Scale for Children—Fourth Edition (WISC-IV) [32], for patients aged between 8 and 16 years, and the Wechsler Adults Intelligence Scale—Fourth Edition (WAIS-IV) for the remaining patients [33]. The group of ADHD patients was evaluated from a clinical point of view using the aforementioned clinical scales, and the cognitive profile was assessed using the cognitive clinical scales previously mentioned while the parents of the ADHD patients completed the CBCL questionnaire.

After 4–6 months, a clinical follow-up was carried out in 45 subjects affected by ASD with an assessment procedure including the clinical measures administered at baseline, as well as the Clinical Global Impression—Improvement Scale (CGI-I) [25], standardized tests to assess Internet use and Internet gaming disorder and CBCL 6–18.

The study conformed to the Declaration of Helsinki. Patients and parents received detailed information on the characteristics of the assessment instruments and treatment options, and all parents provided informed written consent. The methodology of the study was approved by the Regional Ethics Committee for Clinical Trials of Tuscany (Date 27 July 2021, Number 202/2021).

### 2.3. Measures

The Clinical Global Impression—Severity Scale (CGI—Severity) is a scale compiled by the clinician to express a judgment on the severity of the patient’s psychopathology, assessed on the following seven-point scale: 1 = normal, not at all ill; 2 = borderline mentally ill; 3 = mildly ill; 4 = moderately ill; 5 = markedly ill; 6 = severely ill; and 7 = among the most extremely ill patients. This assessment is based on observed and reported symptoms, behavior, and functioning over the past seven days. Because symptom severity may fluctuate over time, the score should reflect the average level of severity over the seven days.

The Children’s Global Assessment Scale (CGAS), adapted from the Global Assessment Scale for adults, is an assessment of functioning aimed at children and young people aged 6 to 17 years. The child or young person is given a single score between 1 and 100, based on the doctor’s assessment of a series of aspects relating to the child’s global and adaptive functioning. Overall functioning is then rated on a scale of 0 to 100, divided into categories.

The Internet Addiction Test (IAT), validated in an Italian version in 2015 [34], is a questionnaire composed of 20 questions measured on a 5-point Likert scale (score 1 for the answer “rarely” and 5 for the answer “always”). The sum of scores between 20 and 49 is considered normal, while scores between 50 and 79 were initially associated with “occasional to frequent” problems, and scores between 80 and 100 were associated with “significant” problems. Kimberly Young, the developer of the IAT, hypothesized a few years ago that the threshold of 80 might be excessively high for identifying adolescents with Internet addiction, and the threshold of 50 was therefore proposed as clinically significant [35]. We therefore considered, consistently with previous studies, that patients with a score above 50 had an Internet addiction (Cronbach’s alpha for the IAT is 0.81) [36].

The Internet Gaming Disorder Scale—Short-Form (IGDS9-SF) [23] is a unidimensional questionnaire that includes 9 questions, which are based on the diagnostic criteria for IGD of the DSM-V. The IGDS9-SF is widely used in IGD research and is supported by numerous psychometric studies carried out on samples of different nationalities [34]. The IGDS9-SF assesses the severity of IGD based on online and offline gaming habits in the last twelve months, with a clinical cut-off of 21 in the Italian version [34] (Cronbach’s alpha 0.76).

The Internet Use, Abuse, and Addiction Questionnaire (UADI) [24] is a validated tool to assess various aspects of Internet addiction for adolescents and young adults, which explores five dimensions: dissociation (tendency to alienate oneself from reality); impact on real life (consequences of Internet use on daily life); experimentation (the use of the Internet as a means of personal and emotional experimentation); addiction (addictive behaviors and/or symptoms such as tolerance, withdrawal, and compulsivity); and escapism (use of the Internet as a strategy to escape daily difficulties). This measure has previously been used to explore Internet use and abuse in a psychiatric population (Cronbach’s alpha for the UADI scale is 0.82.) [4].

The Child Behavior Checklist 6–18 years (CBCL) [37] is a questionnaire composed of 118 items, completed by parents for the evaluation of the behavior of children and adolescents aged between 6 and 18 years, with 8 different scales of syndromic problems (anxiety/depression, withdrawal/depression, somatic symptoms, socialization problems, thinking problems, attention problems, rule-breaking behavior, and aggressive behavior), a total score, an internalizing problems score, and an externalizing problems score. Each item is rated on a 3-level Likert scale, where 0 represents “not true”; 1 is “sometimes or partly true”; and 2 is “often true”.

The Social Responsiveness Scale-2 (SRS-2) [27] is an evaluation scale composed of 65 items, filled in by parents, aimed at evaluating the various symptomatic aspects of autism for the evaluation of children and adolescents from 2 years old and 6 months to 18 years. The items are scored using a 4-level Likert scale (score 1 for the answer “not true” and 4 for the answer “almost always true”). The scale provides a total severity score and also evaluates 5 aspects of the symptomatology of subjects suffering from ASD: “Awareness” (which evaluates the patient’s insight into social difficulties); “Cognition” (which evaluates the ability to understand social situations and/or the tendency to interpret them literally); “Communication” (which specifically concerns communication difficulties); “Motivation” (which evaluates the willingness to place oneself in social situations and the resulting stress if exposed to them); and “Mannerisms” (which evaluates repetitive, stereotyped or bizarre aspects).

The SCQ (Social Communication Questionnaire) [30] is a 40-item questionnaire, completed by parents, which was designed as a screening tool and developed to identify symptoms associated with autism spectrum disorder. The SCQ contains 40 yes/no items to be completed by the parent or primary caregiver. It applies to individuals whose chronological age is more than 4 years, provided that their mental age is more than 2 years.

The Autism Quotient (AQ)—Adolescent Version [28] for children between 12 and 15 years old and the children’s version (AQ—Child Version, for children between 4 and 11 years old) [29] are two questionnaires each composed of 50 items, assessed using a 4-level Likert scale, from “Absolutely agree” to “Absolutely disagree”, aimed at evaluating the patient’s autistic traits. Both scales provide a total score whose cut-off is however different between both versions (in the AQ—Adolescent Version the cut-off is 30, while in the AQ—Child Version the cut-off is 76).

The Systematizing Quotient (SQ) [31] is a questionnaire administered to parents consisting of 55 items, assessed using a 4-level Likert scale, from “Absolutely agree” to “Absolutely disagree”, aimed at evaluating the patient’s autistic traits and, specifically, the aspects of order and systematization. The clinical cut-off for males is 35 and the cut-off for females is 30.

The Wechsler Intelligence Scale for Children—Fourth Edition (WISC-IV) [32] and the Wechsler Intelligence Scale for Adults—Fourth Edition (WAIS-IV) [33] are standardized intelligence scales composed of fifteen subtests, which provide a Total Intelligence Quotient (IQ) and four composite scores or indices, the Verbal Comprehension Index, the Perceptual Reasoning Index, the Working Memory Index, and the Processing Speed Index.

### 2.4. Statistical Analysis

Descriptive statistical procedures (means, standard deviations, ranges, and frequency distributions) were estimated to describe the study sample and assess its clinical characteristics. Statistical analyses were performed with parametric tests (univariate ANOVA test and Student’s *t*-test); post hoc tests were conducted using Bonferroni’s correction. Univariate analysis with Student’s *t*-test for single-paired data on individual clinical groups was used to detect significant changes over time in variables with continuous distribution. Cohen’s ES was calculated for variables with significant differences between groups. Statistical analyses were performed using the SPSS 19.0 program for Windows. The significance level adopted is 0.05 (*p* < 0.05).

## 3. Results

### 3.1. Internet Gaming Disorder in ASD vs. Group of ADHD Patients and Control Group

Through the use of the IGDS9-SF test, among the 77 patients in the ASD group, 35 patients (45.45%) obtained a score equal to or higher than the cut-off of 21, compared with 68 out of 94 (72.34%) in the ADHD patient group and 14 out of 147 (9.52%) in the control group (one-way ANOVA *p* < 0.001) (Table 3).

### 3.2. Comparison between ASD Patients with IGDS9-SF < 21 or ≥21

**Figure 1:**
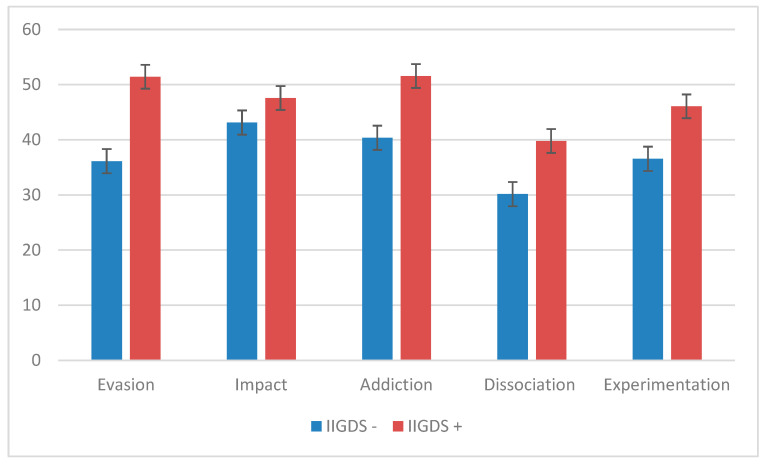
The figure shows the mean scores in the different subscales of the UADI in patients with scores below and above the cut-off at the IGDS9-SF. The clinical cut-off score for the UADI subscales is 50.

Patients with IGDS9-SF scores ≥ 21 (*n* = 35) show significantly higher scores on the IAT test (55.41 ± 12.957, *p* < 0.001, ES = 0.99), as well as on the attention problems subscale of the CBCL (68.94 ± 12.048, *p* = 0.0029, ES = 0.513). It should be noted that neither the functional impairment, assessed by C-GAS, nor the clinical severity, assessed by CGI-S, differ between the groups, just as the various standardized tests regarding autism spectrum disorder symptoms do not differ between the groups, such as the SRS-2, the AQ, the SCQ, and the SQ. Furthermore, the subscales of the WISC-IV showed no significant differences between the two groups (Table 4). Analyzing the two groups with respect to the UADI test, ASD patients with IGDS9-SF equal to or higher than 21 obtain higher scores in the “evasion” subscale (51.43 ± 11.346, *p* < 0.001, ES = 1.472), the “addiction” subscale (51.57 ± 7.144, *p* < 0.001, ES = 1.241), the “dissociation” subscale (39.79 ± 12.771, *p* = 0.021, ES = 0.805), and the “experimentation” subscale (46.07 ± 11.971, *p* = 0.008, ES = 0.937) (Figure 1).

### 3.3. Comparison between ASD and ADHD Patients with IGDS9-SF ≥ 21

ASD patients (*n* = 35) showed a significantly higher mean CGI value, indicative of a more severe clinical situation (4.69 ± 0.583, *p* = 0.003, ES = 0.654) compared with ADHD patients (*n* = 68). Significantly higher scores were found in the ADHD group in the subscales of the CBCL regarding externalizing problems (65.95 ± 9.728, *p* = 0.029, ES = 0.473) and disruptive behavior (69.71 ± 11.925, *p* = 0.021, ES = 0.181). In the group of ASD patients, however, higher scores were found in the subscale of the CBCL regarding withdrawn/depressive aspects (73.59 ± 13.942, *p* = 0.013, ES = 0.538). Finally, we note the presence of slightly lower values on the CGAS scale in the group of ASD subjects, with a tendency to statistical significance (49.86 ± 6.213, *p* = 0.051, ES = 0.416) (Table 5).

### 3.4. Comparison between ASD and ADHD Patients with IGDS9-SF < 21

The ASD patients in this comparison (*n* = 42) showed significantly higher scores on the subscales of the CBCL regarding internalizing problems (68.81 ± 8.477, *p* = 0.003, ES = 0.945), anxious/depressive aspects (69.45 ± 11.01, *p* = 0.021, ES = 0.715), and somatic complaints (62.79 ± 9.251, *p* = 0.036, ES = 0.648) compared with ADHD patients in this group (*n* = 26). We also note the presence of higher CGI values in ASD patients compared with ADHD patients, with a tendency toward significance (4.69 ± 0.729, *p* = 0.051, ES = 0.771) (Table 6).

### 3.5. Data from the Follow-Up Group

The group of patients in the follow-up group included 45 patients diagnosed with ASD, mostly male (37 males, 82.2%, mean age 13.66 ± 2.75 years), who participated in the first phase of the study and also included positive patients and negative patients for one or both tests concerning video addiction (IAT and IGDS9-SF). Patients within this group have undergone drug therapy, rehabilitation, or psychotherapeutic treatment, or all three. The subjects all had a diagnosis of ASD comorbid with other disorders and met the inclusion and exclusion criteria previously mentioned. From the point of view of rehabilitation and pharmacological treatments, 15 patients (33.33%) benefited equally from pharmacological and psychotherapeutic treatment. The most used categories of drugs were methylphenidate in its various formulations (15/45, 33.33%), mood stabilizers (15/45, 33.33%), and atypical antipsychotics (11/45, 24.44%). Finally, 21 out of 45 patients (46.66%) benefited from psychotherapeutic treatment. Within the follow-up group, the patients reported specific comorbidities (Table 7).

In the transition from T0 to T1, 2 patients, equal to 4.44% of the sample, showed a slight worsening, 14 remained stable (31.11%), 24 improved slightly (53.33%) and 5 were moderately improved (11.11%). According to the patient’s global functioning (CGAS), 12 patients out of 45, equal to 26.66%, improved their CGAS score, 12 patients out of 45, equal to 26.66% of the sample, were classified in a better CGAS functioning class and 2 out of 45, equal to 4.44%, are classified in a worse CGAS functioning class.

At the 4–6 month follow-up, patients showed a significant reduction in CGI values from a score of 4.64 ± 0.712 at baseline to a score of 4.22 ± 0.795 at the follow-up (paired *t*-test, t = 4.560, *p* < 0.001, ES = 0.68) and a slight significant increase in CGAS values from a score of 51.51 ± 6.493 at baseline to a score of 54.71 ± 6.704 at the follow-up (paired *t*-test, t = −6.335, *p* > 0.001, ES = 0.944). However, no statistically significant correlations were found regarding the video addiction tests (IAT, IGDS9-SF, and UADI) as well as the evaluation of the syndromic scales of the CBCL 6–18 (Table 8).

## 4. Discussion

Internet addiction and more specifically Internet gaming disorder (IGD) are recently introduced diagnostic categories, often comorbid with other psychiatric disorders [7]. In the literature, ADHD is typically associated with a greater risk of developing IGD [10], although the clinical phenotype of ADHD patients with comorbid IGD is currently poorly described. Similarly, patients with ASD tend to be more prone to excessive use of technological devices [38,39]. However, only one study so far compared the clinical characteristics of IGD in ADHD and ASD pediatric patients [20]. The first objective of the present study was to evaluate IGD prevalence in a sample of pediatric patients with ASD and to compare it with a group of ADHD patients, as well as with a group of healthy controls. Previous studies reported variable rates of prevalence in this clinical population likely because IGD symptoms have been measured by means of multiple instruments and in adult populations [12]. Moreover, we aimed to compare ASD patients with and without comorbid IGD, in order to characterize those who are at higher risk of this behavioral addiction. As already highlighted by other studies in the literature, our study supports the idea that patients with ADHD and ASD have a greater risk of developing IGD, given the greatly higher rate of patients in both groups exceeding the IGDS9-SF cut-off compared with the control population. Specifically, patients with ADHD are those with the highest prevalence of IGD, followed by patients with ASD. The association between IGD and ADHD can be easily explained by the psychological features of ADHD itself, such as the impulsive need for a quick reward, as well as by the tendency toward sensation-seeking behaviors, which are provided by video games and Internet (which is indeed supported by functional MRI evidence of a common neurobiological pattern between the two disorders [40]); moreover, the association between ASD and IGD could be related instead, as already mentioned, both to greater visual skills often present in people with ASD [14] and the possibility to use electronic devices as a possible tool to “facilitate” relationships with others [41]. In order to better clarify this association in patients with ASD, we investigated the clinical and cognitive characteristics of ASD patients with IGDS9-SF scores above the clinical cut-off. Overall, the clinical features of ASD patients with scores above the cut-off on the IGDS9-SF are largely comparable to patients with scores below the cut-off, as are the indices assessed on cognitive tests. Furthermore, no clinically significant differences were found in ASD core symptoms; this evidence was already found in a pediatric population in a 2013 study [20], even if in that case the assessment of autistic symptoms was carried out only by using SCQ, suggesting that the extent of ASD-specific symptoms does not correlate with the presence of IGD. The greatest differences have been found in attention difficulties, investigated using the related syndromic subscale of the CBCL, configuring a phenotype of patients who tend to show a more severe clinical picture. It is also noted that, overall, all the dimensions investigated in the UADI test are highly represented in patients with IGDS9-SF scores higher than the cut-off and, in particular, the dimensions of “dependence” and “evasion”. This evidence was also found in a group of pediatric ADHD patients [42], suggesting that, similarly, patients with ADHD and patients with ASD are more inclined to escape from the real world and dissociate from reality, despite those with addictive and sensation-seeking tendencies being at greater risk of developing IGD. These data are in line with previous studies that showed a marked tendency of ASD patients to use the Internet and video games as a form of escape rather than as a simpler and less anxiety-provoking form of communication [43]. The neuropsychological profile that emerged by comparing the subscales of the WISC-IV and the WAIS-IV showed no differences between the ASD patients who were screened as positive and negative at the IGD standardized tests. This suggests that the cognitive profile does not influence the initial propensity to develop Internet addictions, as found in a pediatric study on ADHD patients [42].

Subsequently, we compared, by using the CGAS and CGI clinical scales and the CBCL 6–18 questionnaire, the group of ASD patients and IGDS9-SF scores above the cut-off with the group of ADHD patients and IGDS9-SF scores above the cut-off; similarly, a comparison was made between the group of ASD patients and IGDS9-SF scores below the cut-off with the group of ADHD patients and IGDS9-SF scores below the cut-off. In both cases, the ASD patient group was, albeit slightly, clinically more severe than the ADHD patient group. In the first comparison, ADHD patients showed higher values in the subscales concerning externalizing problems and disruptive behaviors, while in ASD patients higher scores have been found in the subscale concerning withdrawn/depressive symptoms. In the second comparison, the group of ASD patients presented higher scores in the subscales concerning internalizing problems, anxious/depressive symptoms, and somatic problems. The differences found in the CBCL 6–18 appear, in general, to be attributable to the main clinical condition of the patients, therefore not configuring a peculiar psychopathological profile. It appears important to consider how the various overlaps present within the groups can be partly explained by the frequent comorbidity between ASD and ADHD within the ASD clinical group (but not in the ADHD clinical group), also suggesting the possibility that the characteristics of the two disorders cannot be completely separated and that further studies with larger sample sizes and, especially, with samples of ASD patients without comorbid ADHD could highlight this distinction, which could have relevant implications for identifying personalized therapeutic approaches. In light of these results, in the second phase of the study, we aimed to verify whether psychiatric monitoring and pharmacological and/or psychotherapeutic treatment could be effective in reducing the symptoms of Internet gaming disorder. The results obtained in our sample of ASD patients highlighted a global improvement in the patients’ clinical pictures, assessed using CGAS and CGI. However, no significant improvements were found in the scores of the video addiction tests, nor in the scores assessed on the CBCL 6–18.

However, the generalizability of our findings may be prevented by some limitations in the study design. First, it must be considered that (1) drug prescription was based on the main clinical picture of the patient and not on the presence of IGD, as no specific pharmacological treatments are currently available for this disorder; (2) the small size of our sample leads to less consistency in drawing statistically reliable conclusions; (3) the duration of the follow-up was between 4 and 6 months, thus preventing any improvements to be assessed over a longer period of time; and (4) the number and type of pharmacological and psychotherapeutic treatments and the association between these was particularly heterogeneous, a detail that prevented a direct comparison between the different treatments implemented. Furthermore, the type of study did not allow a comparison with a placebo condition. The wide age range can be also considered as a limitation of the study, as the clinical manifestation of both ASD and ADHD can change over time.

In a similar study conducted on pediatric ADHD patients [42], the “follow-up” variable was associated with a significant reduction in IGD symptoms, while pharmacological treatment did not show the same association. In our sample of ASD patients, the follow-up, although it showed a significant association with improvement from a clinical point of view (measured with CGI) and social adaptability (measured with CGAS), did not show any association with improvement in addiction symptoms. Since the design of the two studies is largely comparable, this difference could be an indication of the fact that the diagnosis of ASD could represent a negative prognostic factor in the long-term regarding Internet gaming disorder compared with ADHD. This conclusion could be also supported by some literature evidence, as specific pharmacological treatments are currently available for ADHD and not for ASD, which have also shown some evidence of effectiveness on IGD symptoms in two studies [44,45], although both studies present some limitations that may raise doubts about the real effectiveness of this pharmacological treatment. These studies, combined with the evidence on the neurobiological mechanisms common between ADHD and IGD [40,46], suggest that if the greater sensitivity to immediate reward mechanisms (not surprisingly stimulated by video games) may be implicated in a greater prevalence of IGD in ADHD patients, the same mechanisms can be a potential target for pharmacological treatments.

Finally, several studies highlight that, concerning addictions to electronic devices [47] and other behavioral addictions [48], no specific “projectiles” or “magic bullets” have been put in place yet [48]; therefore, it is preferable to set up multimodal treatments, based not only on pharmacological but also psychoeducational and psychological therapies [47].

## 5. Conclusions

Internet gaming disorder is closely related to autism spectrum disorder. Dependence on electronic devices identifies ASD patients as a group with higher inattention symptoms. At the same time, however, it must be kept in mind that, as emerged from the data of this study, these patients are particularly affected by both internalizing and externalizing disorders, as well as important comorbidity with ADHD, and that various literature works correlate the presence of IGD with other psychiatric conditions such as social withdrawal, anxiety disorder, depression, OCD and ADHD itself. Therefore, even among those diagnosed with ASD, it is not possible to exclude that the presence of behavioral dependence may be linked not only to the symptoms of the present neurodevelopmental disorder but also to the presence of other comorbid disorders. Based on the data that emerged in the second part of the study, the presence of neuropsychiatric monitoring and pharmacological and/or psychotherapeutic treatment after 4 months determines a significant improvement in the clinical severity and social adaptability of the patient but not in the aspects of video addiction. The latter element emerged during the follow-up of a group of ADHD patients, which could place the diagnosis of ASD as a negative prognostic factor in the follow-up of aspects of video addiction compared with ADHD.

## Figures and Tables

**Table 1:** Baseline clinical characteristics of the ASD group.

	*n*	%
ADHD	55	71.43
Generalized anxiety disorder	45	58.44
Bipolar disorder	35	45.45
Social phobia	26	33.76
Depressive disorder	19	24.67
Obsessive–compulsive disorder	18	23.37
Oppositional defiant disorder	16	20.77
Tic disorder	9	11.68
Learning disorders	7	9.09

**Table 2:** Baseline clinical characteristics of the ADHD group.

	*n*	%
Generalized anxiety disorder	37	39.36
Mood disorder	33	35.1
Bipolar disorder	26	27.65
Oppositional defiant disorder	26	27.65
Tic disorder	11	11.7
Obsessive–compulsive disorder	11	11.7
Depressive disorder	8	8.51

**Table 3:** Prevalence of IGD within patient and control groups.

	IGDS9-SF above the Cut-Off	IGDS9-SF below the Cut-Off	Total
ADHD	68 (72.34%)	26 (27.66%)	94
ASD	35 (45.45%)	42 (54.55%)	77
NC	14 (9.52%)	133 (90.48%)	147

**Table 4:** Comparison between ASD subjects scoring above and below IGDS9-SF cut-off.

Variables	IGDS9-SF above the Cut-Off		IGSF9-SF below the Cut-Off		*p*-Value
	Mean	SD	Mean	SD	
CGAS	49.86	6.213	50.71	7.924	0.604
CGI	4.69	0.583	4.69	0.749	0.976
IAT	55.41	12.957	41.58	14.719	* <0.001
UADI evasion	51.43	11.346	36.12	9.854	* <0.001
UADI impact	47.57	8.528	43.12	10.872	0.195
UADI addiction	51.57	7.144	40.36	9.907	* <0.001
UADI dissociation	39.79	12.771	30.16	11.481	* 0.021
UADI experimentation	46.07	11.971	36.56	9.01	* 0.008
WISC-IV total IQ	96.75	16.352	95.53	16.588	0.828
WISC-IV Verbal Comprehension Index	100.82	19.613	103.19	19.226	0.641
WISC-IV Visual Perception Index	107.67	15.435	107.27	19.146	0.929
WISC-IV Working Memory Index	83.55	19.225	87.62	20.088	0.434
WISC-IV Processing Speed Index	79.08	17.181	82.58	16.405	0.429
SCQ	12.9	6.34	11.61	6.724	0.497
SRS-2 total	72.67	16.201	74.1	12.81	0.763
AQ tot	41.85	25.8	40.5	24.522	0.891
SQ tot	26.86	11.452	28.13	11.922	0.837
CBCL Internalizing Problems	69.21	9.899	68.81	8.477	0.851
CBCL Externalizing Problems	60.91	12.196	57.93	9.322	0.231
CBCL Total	67.26	10.022	65.95	7.942	0.526
CBCL Anxiety/Depression	67.56	11.177	69.45	11.01	0.461
CBCL Withdrawal/Depression	73.59	13.942	69.26	12.68	0.161
CBCL Somatic Complaints	63.79	9.055	62.79	9.251	0.635
CBCL Social Problems	66.21	11.197	66.45	8.609	0.914
CBCL Thought Problems	67.91	9.517	65.67	9.193	0.301
CBCL Attention Problems	68.94	12.048	63.64	8.706	* 0.029
CBCL Aggressive Behavior	60.62	9.41	56.81	7.438	0.052
CBCL Rule-Breaking Behavior	60.55	8.6	60.62	9.41	0.202

Legend: IGDS9-SF: Internet Gaming Disorder Scale–Short-Form; IAT: Internet Addiction Test; UADI: Use, Abuse, and Addiction Questionnaire; C-GAS: Children’s Global Assessment Scale; SCQ: Social Communication Questionnaire; SRS-2: Social Responsiveness Scale-2; AQ: Autism Quotient; SQ: Systematizing Quotient; CBCL: Child Behavior Checklist; WISC-IV: Wechsler Intelligence Scale for Children—Fourth Edition.; *: Statistical significance (*p* < 0.05).

**Table 5:** Comparison between ASD patients and ADHD patients with IGDS9-SF above the cut-off.

Variables	ASD above the Cut-Off		ADHD above the Cut-Off		*p*-Value
	Mean	SD	Mean	SD	
CGAS	49.86	6.213	52.29	5.627	0.051
CGI	4.69	0.583	4.24	0.734	* 0.003
IAT	55.41	12.957	41.58	14.719	0.368
WISC-IV total IQ	96.75	16.352	96.05	13.433	0.862
WISC-IV Verbal Comprehension Index	100.82	19.613	103.37	14.619	0.486
WISC-IV Visual Perception Index	107.67	15.435	103.13	14.818	0.172
WISC-IV Working Memory Index	83.55	19.225	86.67	16.124	0.414
WISC-IV Processing Speed Index	79.08	17.181	82.09	16.509	0.438
CBCL Internalizing Problems	69.21	9.899	65.62	10.5	0.105
CBCL Externalizing Problems	60.91	12.196	65.95	9.728	* 0.029
CBCL Total	67.26	10.022	68.3	9.087	0.606
CBCL Anxiety/Depression	67.56	11.177	66.21	11.051	0.568
CBCL Withdrawal/Depression	73.59	13.942	66.67	12.251	* 0.013
CBCL Somatic Complaints	63.79	9.055	60.52	8.368	0.078
CBCL Social Problems	66.21	11.197	66.67	9.916	0.835
CBCL Thought Problems	67.91	9.517	64.78	10.412	0.148
CBCL Attention Problems	68.94	12.048	70.43	11.014	0.541
CBCL Aggressive Behavior	60.62	9.41	62.24	8.688	0.397
CBCL Rule-Breaking Behavior	60.55	8.6	69.71	11.925	* 0.021

* Statistical significance (*p* < 0.05).

**Table 6:** Comparison between ASD patients and ADHD patients with IGDS9-SF below the cut-off.

Variables	ASD below the Cut-Off		ADHD below the Cut-Off		*p*-Value
	Mean	SD	Mean	SD	
CGAS	50.71	7.924	55.75	4.268	0.088
CGI	4.69	0.749	4.13	0.641	0.051
IAT	41.58	14.719	39.92	11.246	0.628
WISC-IV total IQ	95.53	16.588	98	14.663	0.619
WISC-IV Verbal Comprehension Index	103.19	19.226	104.26	13.965	0.822
WISC-IV Visual Perception Index	107.27	19.146	102.26	15.864	0.307
WISC-IV Working Memory Index	87.62	20.088	87.17	18.975	0.935
WISC-IV Processing Speed Index	82.58	16.405	89.95	16.576	0.115
CBCL Internalizing Problems	68.81	8.477	60.14	11.086	* 0.003
CBCL Externalizing Problems	57.93	9.322	60.2	13.597	0.478
CBCL Total	65.95	7.942	63.2	11.092	0.306
CBCL Anxiety/Depression	69.45	11.01	61.87	9.365	* 0.021
CBCL Withdrawal/Depression	69.26	12.68	62.87	11.141	0.09
CBCL Somatic Complaints	62.79	9.251	57.2	6.416	* 0.036
CBCL Social Problems	66.45	8.609	62.33	9.309	0.125
CBCL Thought Problems	65.67	9.193	61.07	10.787	0.118
CBCL Attention Problems	63.64	8.706	65.33	7.148	0.503
CBCL Aggressive Behavior	56.81	7.438	60.6	9.038	0.115
CBCL Rule-Breaking Behavior	60.62	9.41	63.6	10.96	0.278

* Statistical significance (*p* < 0.05).

**Table 7:** Baseline clinical characteristics of the follow-up group.

	*n*	%
ADHD	34	75.55
Generalized anxiety disorder	24	53.33
Bipolar disorder	23	51.11
Social phobia	16	35.55
Oppositional defiant disorder	12	26.66
Depressive disorder	11	24.44
Obsessive–compulsive disorder	7	15.55
Learning disorders	4	8.88
Tic disorders	3	6.66

**Table 8:** Difference between T0 and T1 in the measures of assessment.

Variables	Mean	SD	t	DF	*p*-Value
CGAS–CGAS 1	−3.2	3.388	−6.335	44	* <0.001
CGI–CGI 1	0.422	0.621	4.56	44	* <0.001
IAT–IAT 1	−0.703	13.01	−0.329	36	0.372
IGDS9-SF–IGDSF9 1	0.784	7.215	0.661	36	0.513
UADi Ev–UADI Ev 1	1.294	11.741	0.454	16	0.656
UADI Imp–UADI Imp 1	−2.529	8.338	−1.251	16	0.229
UADI Add–UADI Add 1	2.765	6.088	1.872	16	0.08
UADI Dis–UADI Dis 1	1.588	7.867	0.832	16	0.417
UADI Exp–UADI Exp 1	−0.765	5.203	−0.606	16	0.553
CBCL Int–CBCL Int 1	0.892	6.527	0.831	36	0.411
CBCL Ext–CBCL Ext 1	1.405	6.825	1.253	36	0.218
CBCL Tot–CBCL Tot 1	1.243	6.18	1.224	36	0.229
CBCL Anx/Dep–CBCL Anx/Dep 1	0.676	8.541	0.481	36	0.633
CBCL With/Dep–CBCL With/Dep 1	2.649	10.163	1.585	36	0.122
CBCL Som–CBCL Som 1	0.514	6.731	0.464	36	0.645
CBCL Soc–CBCL Soc 1	−0.811	6.749	−0.731	36	0.47
CBCL Tho–CBCL Tho 1	0.378	7.577	0.304	36	0.763
CBCL Att–CBCL Att 1	0.541	8.325	0.395	36	0.695
CBCL Agg–CBCL Agg 1	0.919	5.074	1.102	36	0.278
CBCL Rule–CBCL Rule 1	1.243	7.112	1.063	36	0.295

* Statistical significance (*p* < 0.05).

## Data Availability

The data presented in this study are available upon request from the corresponding author. The data are not publicly available due to containing information that could compromise the privacy of research participants.

## Supplementary Materials

The following supporting information can be downloaded at: https://www.mdpi.com/article/10.3390/brainsci14020154/s1, Supplementary Figure S1.
