# Supplementary figures and images for: Internet Gaming Disorder in Children and Adolescents with Autism Spectrum Disorder and Attention Deficit Hyperactivity Disorder
Source: Brain Sci. 2024 Feb 2;14(2):154. doi: 10.3390/brainsci14020154 (PMC10887068; doi:10.3390/brainsci14020154)

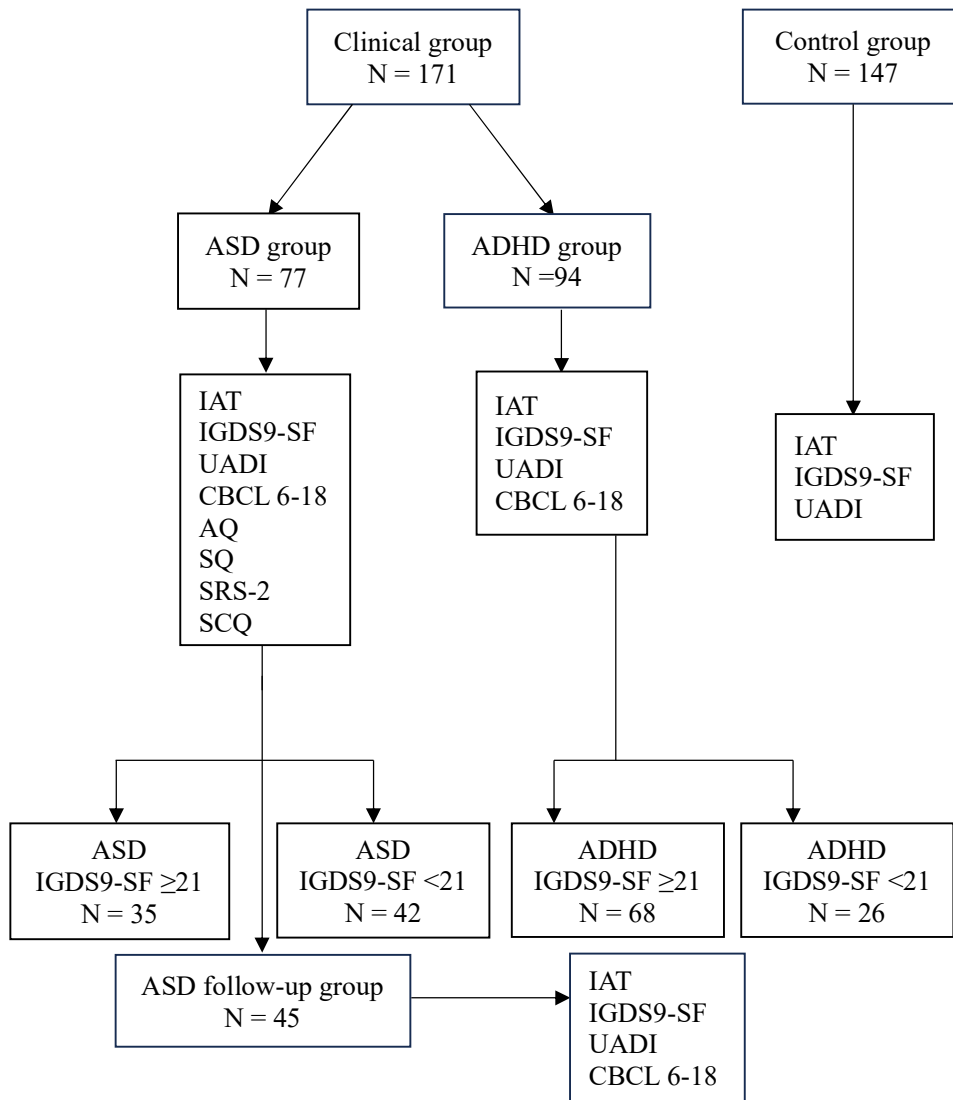

Supplement: Supplementary file 1 [file brainsci-14-00154-s001.zip › brainsci-2813679-supplementary.pdf]
